# Exploring the Human Cytomegalovirus Core Nuclear Egress Complex as a Novel Antiviral Target: A New Type of Small Molecule Inhibitors

**DOI:** 10.3390/v13030471

**Published:** 2021-03-12

**Authors:** Sewar Alkhashrom, Jintawee Kicuntod, Sigrun Häge, Johannes Schweininger, Yves A. Muller, Peter Lischka, Manfred Marschall, Jutta Eichler

**Affiliations:** 1 Division of Medicinal Chemistry, Department of Chemistry and Pharmacy, University of Erlangen-Nürnberg (FAU), 91058 Erlangen, Germany; sewar.alkhashrom@fau.de; 2 Institute for Clinical and Molecular Virology, University of Erlangen-Nürnberg (FAU), 91054 Erlangen, Germany; jintawee.kicuntod@extern.uk-erlangen.de (J.K.); sigrun.haege@fau.de (S.H.); manfred.marschall@fau.de (M.M.); 3 Division of Biotechnology, Department of Biology, University of Erlangen-Nürnberg (FAU), 91052 Erlangen, Germany; johannes.schweininger@fau.de (J.S.); yves.muller@fau.de (Y.A.M.); 4 AiCuris Anti-Infective Cures GmbH, 42117 Wuppertal, Germany; peter.lischka@aicuris.com

**Keywords:** human cytomegalovirus, nuclear egress complex (NEC), core NEC inhibitor, antiviral activity, small molecule inhibitor

## Abstract

Nuclear egress is an essential process in the replication of human cytomegalovirus (HCMV), as it enables the migration of newly formed viral capsids from the nucleus into the cytoplasm. Inhibition of the HCMV core nuclear egress complex (core NEC), composed of viral proteins pUL50 and pUL53, has been proposed as a potential new target for the treatment of HCMV infection and disease. Here, we present a new type of small molecule inhibitors of HCMV core NEC formation, which inhibit the pUL50-pUL53 interaction at nanomolar concentrations. These inhibitors, i.e., verteporfin and merbromin, were identified through the screening of the Prestwick Chemical Library^®^ of approved drug compounds. The inhibitory effect of merbromin is both compound- and target-specific, as no inhibition was seen for other mercury-organic compounds. Furthermore, merbromin does not inhibit an unrelated protein–protein interaction either. More importantly, merbromin was found to inhibit HCMV infection of cells in three different assays, as well as to disrupt HCMV NEC nuclear rim formation. Thus, while not being an ideal drug candidate by itself, merbromin may serve as a blueprint for small molecules with high HCMV core NEC inhibitory potential, as candidates for novel anti-herpesviral drugs.

## 1. Introduction

Human cytomegalovirus (HCMV) is a ubiquitous human β-herpesvirus with a seroprevalence ranging from 40% to 95% in various regions of the world, establishing a life-long latent infection. While HCMV infection is typically asymptomatic in immunocompetent individuals, immunonaïve hosts, as well as immunocompromised patients with stem cell or solid organ transplantation, AIDS, or cancer, often develop severe symptoms upon HCMV infection, such as fever, fatigue, muscle aches, enlarged lymph nodes, and sore throat [1]. More importantly, congenital HCMV infection acquired during pregnancy frequently results in severe cytomegalovirus inclusion disease and developmental defects, such as sensorineural hearing loss (SNHL) and mental retardation or microcephaly, in the neonate [2]. Currently available drugs for the treatment of HCMV infections include ganciclovir, its oral prodrug valganciclovir, cidofovir, and foscavir, which all target the viral DNA polymerase. While currently available anti-HCMV drugs are of great benefit for the management of HCMV infections in immunocompromised patients, they are also associated with undesirable side effects, poor oral bioavailability, and modest efficacy, as well as the development of virus drug-resistance [1]. More recently, letermovir, which targets the HCMV-specific terminase complex, has been approved for the prevention of HCMV infection and reactivation, albeit for a limited patient population, i.e., hematopoietic stem cell transplant recipients [3]. In view of the still limited number of options for the treatment of HCMV infections, the search for novel therapeutic strategies, including the exploration and validation of new drug targets in the virus entry and replication process, continues to be a strong biomedical and clinical research focus [3,4,5,6].

Replication of HCMV involves a process termed nuclear egress, which enables migration of the newly formed viral capsids from the nucleus into the cytoplasm, and which entails a range of precisely tuned protein–protein interactions [7]. The formation of the core nuclear egress complex (core NEC), composed of viral proteins pUL50 and pUL53, which is anchored to the inner nuclear membrane, is a central component of nuclear egress, as it serves as a scaffold for the assembly of a multimeric NEC composed of viral and cellular proteins [8,9]. Extensive structural analysis of the pUL50-pUL53 interaction, through x-ray crystallography of the protein complex, has provided detailed information on the structural features of the proteins, as well as the protein interface [10,11,12,13,14]. While both proteins adopt a globular fold with mixed secondary structure elements, the hallmark element of the pUL50-pUL53 interaction is an N-terminal α-helical, hook-like extension in pUL53, which is independent of the overall globular protein fold, and contributes approximately 80% to the total interface area with pUL50 [10]. Interestingly, the pUL50-pUL53 heterodimeric complex partially assembles into hexameric ring-like structures [10], and is also able to oligomerize in vitro [11]. We have recently shown that a 29-mer synthetic peptide presenting the pUL53 N-terminal hook specifically interacts with soluble pUL50, i.e., it does not bind to BFRF1, which is the Epstein–Barr virus homolog of pUL50 [12,13]. Furthermore, the pUL53 hook peptide is able to inhibit the pUL50-pUL53 interaction in vitro at submicromolar concentrations. A similar N-terminal pUL53 peptide was previously shown to interfere, albeit at 100-fold higher concentrations, with the pUL50-pUL53 interaction [15]. Based on these data, we have proposed inhibition of the pUL50-pUL53 interaction as a novel antiviral strategy against HCMV infections. Due to its size (molecular weight: approximately 4 kDa), as well as its susceptibility to proteolytic degradation, however, the pUL53 hook peptide is a less than ideal candidate for inhibition of the pUL50-pUL53 interaction in vivo. This becomes even more apparent considering that a successful inhibitor of this interaction has to be taken up by the cell, as well as to penetrate the nuclear membrane. Based on these considerations, small molecule inhibitors would be more adequate, as they are more likely to passively pass through membranes. On the other hand, targeting protein–protein interactions with small molecules is generally thought to be challenging, due to the larger, more flat interfaces involved in these interactions, as compared with the defined binding pockets of other drug targets, such as receptors and enzymes. It should be noted, however, that small molecules have been shown to indirectly interfere with protein–protein interactions, e.g., by disrupting structural elements in the proteins that are essential for the interaction [16]. This can be achieved through binding of the small molecule to so-called allosteric binding sites of the proteins that are not involved in the protein interface [17].

Therefore, we set out to search for small molecule inhibitors of the HCMV pUL50-pUL53 interaction as a blueprint for a novel antiviral strategy based on disrupting viral nuclear egress. As a source for such potential inhibitors, we selected the Prestwick Chemical Library^®^ (https://www.prestwickchemical.com/screening-libraries/prestwick-chemical-library/, accessed on 3 February 2021), which is composed of a total of 1520 off-patent, approved, small molecule drug compounds, presenting a high chemical and pharmacological diversity, and addressing more than 400 drug targets. As these compounds, in order to be approved for clinical use, have undergone thorough pre-clinical and clinical evaluation, re-purposing them for alternative medical indications is expected to require less extensive studies, as compared with de novo designed compounds, facilitating their approval. 

## 2. Materials and Methods 

### 2.1. pUL50-pUL53 Inhibition Assay

Recombinant pUL50 and pUL53 were produced and purified as previously described [12]. High binding Immulon microtiter plates were coated with 100 µL pUL53 (1 µg/mL) in sodium carbonate buffer pH 9.5, overnight at 4 °C. After blocking with 200 µL 1% BSA in 0.1 M phosphate buffer, pH 7.2, for two hours, plates were incubated with 50 µL library compound/mixture in serial dilutions, starting at 1.25 µM, together with 50 µL His-tagged pUL50 (0.25 µg/mL) for two and a half hours. His-tagged pUL50 was detected using anti-His-HRP (Merck, Darmstadt, Germany, 1:40,000). All proteins and antibodies were in 0.1 M phosphate buffer, pH 7.2, containing 0.1% BSA and 0.01% Tween 20. Plates were washed four times with 0.01% Tween 20 in 0.1 M phosphate buffer, pH 7.2, after each incubation step. Plates were developed with *o*-phenylenediamine (OPD) (1 mg/mL) in the presence of 0.03% H_2_O_2_ for approximately 5 min in the dark. After the reaction was stopped with 2 M H_2_SO_4_, absorbance was read at 492 nm. IC_50_ values were determined from the % inhibition data using the program GraphPad. Inhibition was calculated according to the following formula:

(1) $$\%\;Inhibition\;=\;\left[\;1\;-\;\left(Asample\;-\;Ablank1\right)/\;\left(A100\%\;-\;Ablank2\right)\right]\;\times \;100$$

in which “100%” is a sample without inhibitor, “blank1” is a sample without pUL53, and “blank2” is a sample without pUL53 and without inhibitor.

### 2.2. HIV-1 gp120–mAb 447-52D Inhibition Assay

High binding Costar microtiter half area plates were coated with 100 µL gp120_HxBc2_ (Immune Technology, New York, NY, 0.5 µg/mL) in sodium carbonate buffer pH 9.5, overnight at 4 °C. After blocking with 1% BSA in 0.1 M phosphate buffer, pH 7.2, for two hours, plates were incubated with the CXCR4 mimetic peptide CX4M1 and merbromin, respectively, in serial dilutions, starting at 60 µM, together with mAb 447-52D, obtained through the NIH AIDS Research and Reference Reagent Program (0.2 µg/mL) for 3 h. Bound antibody was detected using anti-human IgG-HRP (Merck, Darmstadt, Germany, 1:10,000). All proteins and antibodies were in 0.1 M phosphate buffer, pH 7.2, containing 0.1% BSA and 0.01% Tween 20. Plates were washed four times with 0.01% Tween 20 in 0.1 M phosphate buffer, pH 7.2, after each incubation step. Plates were developed, and IC_50_ values calculated, as described above (Section 2.1).

### 2.3. Cytotoxicity Assay

Human foreskin fibroblast (HFF) cells were cultivated in a 96-well plate with a density of 1.35 × 10^4^ cells per well and incubated at 37 °C for 24 h. The cultured HFF cells were treated with merbromin and verteporfin, respectively, at concentrations ranging from 1.56 to 100 µM, and incubated at 37 °C for seven days. A sample without compound (DMSO) served as a negative control, and a sample with 1 µM staurosporine (STP) as a positive control. On day 7, neutral red solution (40 µg/mL, Merck, Darmstadt, Germany, N2889) was added to cultivated cells and incubated at 37 °C for 2–4 h, followed by addition of destaining solution (ethanol/water/acetic acid, 50:49:1). Fluorescence was read at excitation/emission at 560/630 nm to quantify the uptake of neutral red.

### 2.4. Virus Infection, Plaque Reduction, and Viral Yield Assays

Primary human foreskin fibroblasts (HFFs, own repository of primary cell cultures) were propagated as previously described [18]. Then, 2 × 10^5^ HFFs were seeded in 12-well plates for 1 day prior to infection with HCMV AD169 at a multiplicity of infection (MOI) of 0.1. After 90 min of viral absorption, cells were incubated with merbromin at concentrations ranging from 0.3 to 10 µM at 37 °C for 5 days. Viral supernatants were collected at 5 days post infection (d.p.i.). For the viral yield assay, HFFs were incubated with a serial dilution of viral supernatants at 37 °C for 1.5 h. Subsequently, cells were overlaid with 2 mL of a 1:1 mixture of 0.6% agarose and medium (MEM containing 2× GlutaMAX^TM^, 20 μg/mL gentamicin, and 20% fetal bovine serum). For the plaque reduction assay, cells infected with HCMV AD169 at MOI 0.1 were overlaid with the 1:1 mixture of agarose:medium containing merbromin at concentrations ranging from 0.3 to 10 µM. In both assays, cells were further incubated at 37 °C for 5 to 7 days, followed by staining with 1% crystal violet in 20% EtOH to visualize viral plaque formation. Plaques were counted under a light microscope. In both assays, a sample containing DMSO instead of merbromin served as a negative control.

### 2.5. Quantitative Polymerase Chain Reaction (qPCR)

The viral genome equivalents of the supernatants of HCMV-infected HFFs treated with merbromin at concentrations ranging from 0.3 to 10 µM were quantified by qPCR. The supernatants were centrifuged at 1500× *g* and digested with proteinase K for 1 h at 56 °C to release viral particles. The reactions were stopped at 95 °C for 5 min. The amount of extracellular viral genomic loads was measured in 5 mL of each sample by real-time PCR (TaqMan-PCR). Two primers, namely 5’CMV (AAGCGGCCTCTGATAACCAAG) and 3’CMV (GAGCAGACTCTCAGAGGATCGG), which anneal to a sequence within the major immediate early gene region of HCMV, were utilized to amplify and quantify the viral genome. In addition, an FAM/TAMRA-labeled probe was used for detection. The viral load of a sample treated with DMSO only served as a negative control. HCMV genome equivalents from merbromin-treated viral supernatants were calculated as % of the negative control. 

### 2.6. Indirect Immunofluorescence Assay and Confocal Laser-Scanning Microscopy

HFFs were cultivated in 6-well plates on cover slips, and used for HCMV infection at MOI of 0.1. After 90 min of viral absorption, cells were treated with the indicated concentrations and incubated at 37 °C for 5 days. On day 5, cells were fixed with 10% formalin (8 min, room temperature). Afterwards, HFFs were permeabilized using 0.2% Triton X-100 in PBS and were blocked with cohn II. Cells were incubated with the indicated primary antibodies for 60 min at 37 °C prior to double staining with secondary antibodies conjugated with Alexa Fluor^®^ 555 and Alexa Fluor^®^ 647. The nucleus was counterstained with DAPI Vectashield mounting medium. Data for immunofluorescence were collected using a TCS SP5 confocal laser-scanning microscope (Leica Microsystems, Wetzlar, Germany). Images of a confocal plane were taken with a line average of 3 at a magnification of 1664 × 1664. 

### 2.7. Antibodies

Monoclonal (mAb) and polyclonal (pAb) antibodies were used to detect the following cellular proteins: rabbit mAb-lamin A/C (EPR4100, Abcam), mouse mAb-IE1p72 (P63-27), mouse mAb-pp28, and mouse mAb-UL53.01 (kindly provided by Stipan Jonjic and Tihana Lenac Rovis, University of Rijeka, Croatia). Alexa Fluor 555- and 647-conjugated antibodies were used as secondary antibodies for indirect immunofluorescence staining (Molecular Probes, Eugene, OR, USA). 

## 3. Results and Discussion 

### 3.1. Identification of HCMV Core NEC Inhibitors from the Prestwick Chemical Library^®^

**Figure 1:**
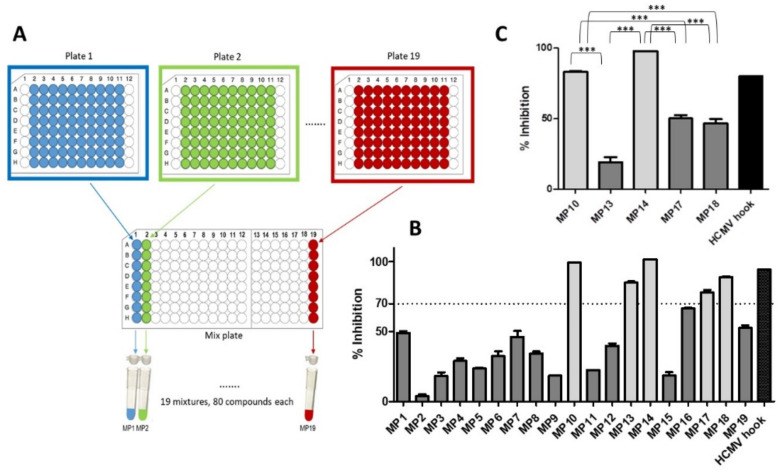
Screening of the Prestwick Chemical Library^®^ for inhibition of the human cytomegalovirus (HCMV) pUL50-pUL53 interaction. (**A**) Scheme of pooling the library compounds into 19 mixtures (MP1 through MP19). (**B**) Inhibition of the HCMV pUL50-pUL53 interaction by the 19 mixtures, as well as the peptide presenting the N-terminal helical hook of HCMV pUL53 (HCMV hook) at 1.25 µM/compound. (**C**) Inhibition of the HCMV pUL50-pUL53 interaction by selected mixtures at 0.42 µM/compound. Error bars present SEMs of at least two experiments. Statistical significance was calculated using the ANOVA test with a subsequent Bonferroni’s multiple comparison test. *P*-values ≤ 0.05 were considered significant, as indicated by ***. See Section 2.1 for experimental detail.

Testing a total of 1520 compounds for any kind of biological activity clearly requires the availability of a parallel assay format. We have recently developed a parallel pUL50-pUL53 inhibition assay [12] (see Section 2.1 for details), which is performed in 96-well plates, and which can therefore be readily used for library screening. In order to simplify the screening procedure, the library compounds were pooled into mixtures, rather than testing each of the 1520 library compounds individually. The library compounds were provided as 10 mM solutions in DMSO, organized in 19 96-well plates, with 80 compounds to each plate. For the first screening round, the compounds of each plate were pooled, generating 19 mixtures (MP1 through MP19) (Figure 1A), each containing 80 compounds, with each compound being present at 125 µM (10 mM / 80). The 19 mixture solutions were further diluted in assay buffer to a concentration of 1.25 µM/compound, generating solutions that contained only 1% DMSO, which were then tested in the pUL50-pUL53 inhibition assay. The peptide presenting the HCMV pUL53 N-terminal helical hook fragment (HCMV hook) served as a positive control. Five of the 19 library mixtures (MP10, MP13, MP14, MP17, and MP18) were found to inhibit the pUL50-pUL53 interaction by more than 70% (Figure 1B). These five mixtures were then tested at a lower concentration (0.42 µM), resulting in the identification of MP10 and MP14 as the mixtures with the strongest inhibitory activity (Figure 1C).

**Figure 2:**
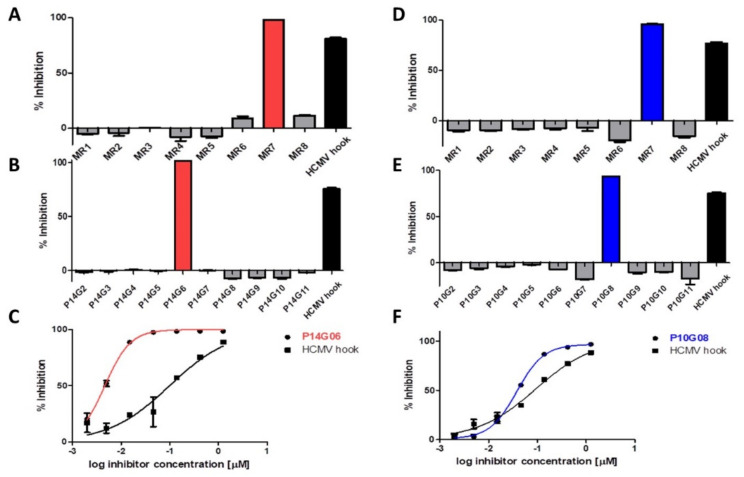
Identification of the most active inhibitors of the pUL50-pUL53 interaction from plate mixtures MP14 (left) and MP10 (right). (**A**,**D**). Screening of the row mixtures of plate 14 (**A**) and plate 10 (**D**). (**B**,**E**) Inhibition of the pUL50-pUL53 interaction by the individual compounds making up the most active row mixtures of plate 14 (**B**) and plate 10 (**E**). (**C**,**F**) Dose-dependent inhibition of the pUL50-pUL53 interaction by the most active inhibitors (P14G06 and P10G08), compared with the peptide presenting the N-terminal helical hook of HCMV pUL53 (HCMV hook). Error bars present SEMs of at least two experiments. See Section 2.1 for experimental detail.

Plate mixtures MP10 and MP14 were selected for further evaluation. Combining the compounds of each row of plate 14, eight new mixtures (P14MR1 through P14MR8) of 10 compounds each were generated and tested at 0.42 µM/compound, which resulted in the identification of P14MR7 as the most active mixture (Figure 2A). Finally, testing the 10 component compounds of mixture P14MR7 (P14G02 through P14G11) yielded compound P14G06 as the most active inhibitor of the pUL50-pUL53 interaction (Figure 2B). Notably, the inhibitory activity of this compound (IC_50_ = 4 nM) is approximately 25-fold stronger than that of the pUL53 hook peptide (IC_50_ = 98 nM) (Figure 2C), and thus well in the range of activities required for potential therapeutic applications. According to the provided library documentation, compound P14G06 is identical to verteporfin. This drug is used as a photosensitizer for photodynamic therapy to eliminate abnormal blood vessels in the eye, which are associated with conditions such as the wet form of macular degeneration [19]. Recently, verteporfin has also been shown to inhibit the function of the oncogene YAP1 through regulation of YAP1 SUMOylation, proposing verteporfin for clinical use to treat breast cancer [20], as well as myeloma [21].

The process of identifying the most active individual compounds was then repeated for plate 10 (Figure 2D,E), resulting in the identification of compound P10G08 as the most potent inhibitor of the pUL50-pUL53 interaction (IC_50_ = 38 nM, Figure 2F). This compound is identical to merbromin, an organomercuric compound, which was formerly used as a topical antiseptic to treat minor wounds, burns, and scratches [22], as well as for the antisepsis of the umbilical cord and wounds with inhibited scar formation, such as neuropathic ulcers and diabetic foot sores [23]. Due to the well-established toxicity of mercury compounds, however, clinical use of merbromin has largely been discontinued in the United States and most European countries. Considering these circumstances, it appears less promising to view merbromin as a candidate for new therapeutic approaches. It may, however, serve as a starting structure, which can be chemically modified to alleviate the toxicity, while maintaining the high core NEC inhibitory activity.

In summary, screening of the Prestwick Chemical Library^®^ resulted in the identification of two drug compounds, i.e., verteporfin and merbromin, which were shown to exhibit nanomolar inhibitory activity against the HCMV pUL50-pUL53 interaction. This was possible using a very robust, parallel in vitro inhibition assay involving recombinant proteins, in conjunction with a compound pooling strategy that enabled the straightforward identification of the two candidate compounds within the library of 1520 compounds. It should be noted that these two compounds are highly unlikely to have been identified as HCMV core NEC inhibitors using alternative approaches, illustrating the utility of library approaches for the de novo identification of bioactive compounds. Ongoing and future studies include the chemical modification of merbromin with the aim of eliminating its toxicity, as well as the search for more small molecule inhibitors using additional compound libraries. Furthermore, analysis of the molecular mechanism of the inhibitory effect of merbromin will include the identification of its binding partner (pUL50 or pUL53), as well as the binding site, through co-crystallization with the respective protein, and subsequent analysis of the complex crystal structures. 

### 3.2. Cytotoxicity and Selectivity of Merbromin

**Figure 3:**
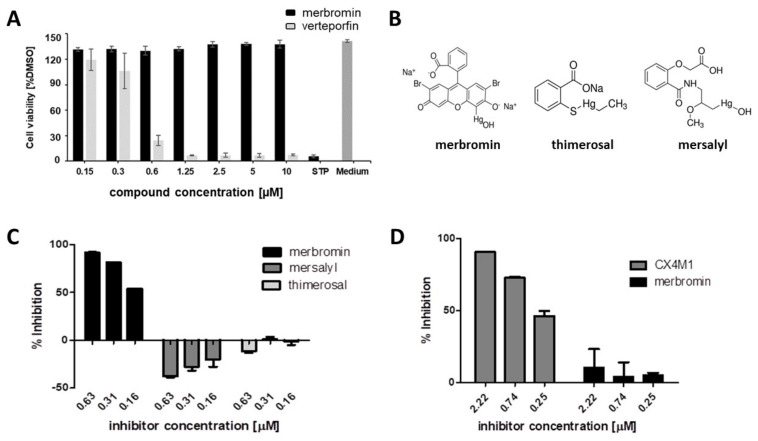
Cytotoxicity and selectivity of merbromin. (**A**) Cytotoxicity of merbromin and verteporfin. (**B**) Chemical structures of merbromin, thimerosal, and mersalyl. (**C**) Effect of merbromin, thimerosal, and mersalyl o the HCMV pUL50-pUL53 interaction. (**D**) Effect of the CXCR4 mimetic peptide CX4M1 and merbromin on the HIV-1 gp120–mAb 447 52D interaction. Error bars present SDs (**A**) and SEMs (**B**–**D**), respectively, of at least two experiments. See Section 2.3 (**A**), Section 2.1 (**B**) and Section 2.2 (**D**) for experimental detail.

**Figure 4:**
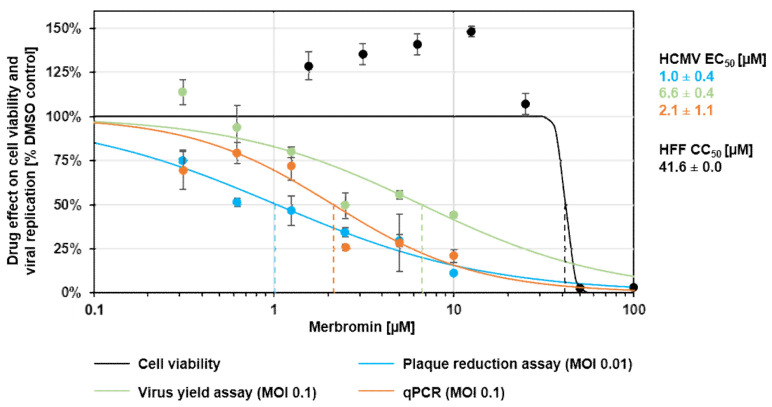
Anti-HCMV activity and lack of cytotoxicity (black curve) of merbromin in the plaque reduction assay (blue curve), virus yield assay (green curve), and genome-specific qPCR assay (orange curve). Error bars present standard deviations calculated from three or four different experiments. See Section 2.3, Section 2.4 and Section 2.5 for experimental detail.

As both verteporfin and merbromin have been associated with a considerable degree of toxicity, we addressed the in vitro cytotoxicity of both compounds, prior to testing them in cell-based HCMV infection assays. Interestingly, verteporfin, but not merbromin, was shown to be toxic to human foreskin fibroblast (HFF) cells at 0.6 µM (Figure 3A), while no cytotoxicity was found for merbromin, even at concentrations as high as 25 µM Figure 3A and Figure 4). Therefore, only merbromin was selected for subsequent evaluation of its selectivity, as well as antiviral activity. 

Binding of mercury to cysteine residues in proteins mediates multiple toxic effects, in particular inhibition of enzymes and other proteins that contain free cysteine residues that are not involved in disulfide bridges [24]. Consequently, it appeared well possible that the inhibitory effect of merbromin on the pUL50-pUL53 interaction was not specific to merbromin, and that other mercury-containing compounds would have similar effects. Therefore, we tested two different mercury compounds, i.e., thimerosal and mersalyl, for inhibition of the pUL50-pUL53 interaction. Thimerosal is used as a preservative in pharmaceutical products, including ophthalmic solutions, otic drops, topical medicine, and vaccines, to protect them from microbial degradation [25]. Mersalyl, on the other hand, is a diuretic drug that is no longer in clinical use [26]. Closer inspection of the chemical structures of all three compounds (Figure 3B) reveals that mersalyl is more closely related to merbromin than thimerosal, as both merbromin and mersalyl contain an R-Hg-OH moiety, while the mercury atom in thimerosal is not bound to a hydroxyl group, but to a substituted thiol (R-Hg-S-R’). Regardless of these chemical features, neither thimerosal nor mersalyl is able to inhibit the pUL50-pUL53 interaction (Figure 3C), indicating that the inhibitory activity of merbromin is not solely due to its mercury atom, but that the chemical scaffold of the compound, i.e., a benzoic acid bound to a xanthene ring, plays a role as well. 

The target selectivity of merbromin was addressed by testing its ability to interfere with an unrelated protein–protein interaction, i.e., the interaction of HIV-1 gp120 with the monoclonal antibody 447 52D, which recognizes the gp120 V3loop [27]. Unlike the CXCR4 mimetic peptide CX4M1 [28], which, similar to the HIV-1 coreceptor CXCR4, binds to the gp120 V3 loop [29], merbromin does not inhibit the gp120–447 52D interaction (Figure 3D), indicating a target selectivity of the inhibition of the pUL50-pUL53 interaction. In summary, based on the established compound and target selectivity, it can be concluded that the inhibitory activity of merbromin is not simply based on an unspecific interaction of the merbromin mercury with the proteins, but a specific effect of merbromin on the pUL50-pUL53 interaction.

### 3.3. Antiviral Activity of Merbromin

To address the question of whether the inhibitory activity of merbromin on the pUL50-pUL53 interaction in vitro translates into an antiviral activity of the compound, it was tested in a standard plaque reduction assay (PRA) in conjunction with human foreskin fibroblast (HFF) cells and HCMV AD169 at an MOI of 0.01 (Figure 4). In this assay, merbromin was shown to dose-dependently inhibit HCMV intracellular replication and plaque formation, with an EC_50_ value of 1.0 ± 0.4 µM (Figure 4, blue curve), without visible adverse effects on cell morphology or viability. In addition to the plaque reduction assay, merbromin was also tested in a virus yield assay that measures the production and release of infectious virus. The inhibitory activity of merbromin in this assay (EC_50_ = 6.6 ± 0.4 µM; Figure 4, green curve) was similar to that in the PRA assay. Finally, the antiviral effect of merbromin could also be confirmed by highly sensitive HCMV-specific qPCR (EC_50_ = 2.1 ± 1.1 µM; Figure 4, orange curve). In summary, the anti-HCMV activity of merbromin could be demonstrated in three different experimental settings, while the level of cytotoxicity remained very low, with a CC_50_ value of 41.6 ± 0.0 µM (Figure 4, black curve), i.e., cell viability was not affected by merbromin at concentrations relevant to antiviral activity. 

### 3.4. Selective Effect of Merbromin on the HCMV Core NEC

**Figure 5:**
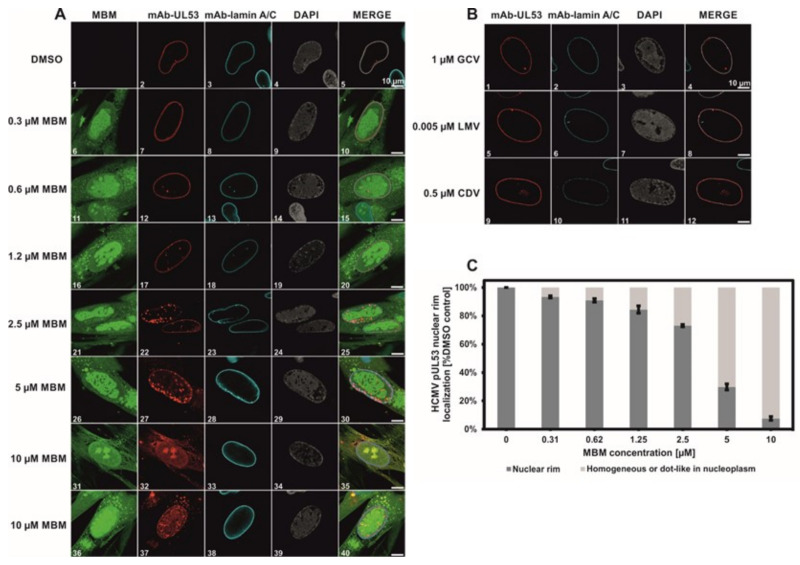
Merbromin (MBM, (**A**)), but not GCV, LMV, and CDV (**B**), interfere with HCMV nuclear egress complex (NEC) nuclear rim formation in HCMV-infected human foreskin fibroblasts (HFFs). (**A**). Confocal imaging (indirect immunofluorescence staining) of viral pUL53 and cellular lamin (**A**/**C**). Counterstainings of the autofluorescent merbromin and the nuclei (DAPI) are shown, and a merge of all signals is given. (**C**). Quantitation of the concentration-dependent inhibition of normal pUL53 nuclear rim localization by merbromin. Error bars present SDs of three experiments. Scale bars indicate 10 µm. See Section 2.6 for experimental detail.

Having established an HCMV-inhibitory activity of merbromin, we then set out to explore the mechanism of this effect. Based on the previously demonstrated inhibition of the pUL50-pUL53 interaction by merbromin, we hypothesized that the compound should be able to interfere with NEC-typical nuclear rim localization in HCMV-infected fibroblasts, which is regulated by the pUL50-pUL53 interaction [13,30]. For this purpose, HFFs were infected with HCMV at an MOI of 0.1 and fixed at 5 days p.i. for indirect immunofluorescence stainings (Figure 5). Colocalization of viral pUL53 with cellular lamin A/C was used as an indicator of viral core NEC formation. Here, we could show that merbromin dose-dependently effected a reduction in the number of HCMV-positive cells, as well as an alteration of nuclear rim NEC formation, with increasingly pronounced intranuclear dot-like speckling of pUL53, within the HCMV-positive cell population (Figure 5A, panels 16–35). This effect ultimately resulted in complete abrogation of rim-like NEC localization, yielding a homogeneous staining pattern of pUL53 in the entire nucleoplasm, in the presence of 10 µM merbromin (Figure 5A, panels 31–40). Interestingly, this effect of merbromin was not seen for the known and approved HCMV drugs ganciclovir (GCV), letermovir (LMV), and cidofovir (CDV) (Figure 5B; drug concentrations were adjusted to their individual EC_50_ values). These drugs are known to address targets other than the viral core NEC, i.e., the viral DNA polymerase (GCV and CDV) and the HCMV terminase complex (LMV), respectively. Hence, the effect of merbromin on pUL53 localization appears to be specific to this compound. Quantitation of this effect through microscopic counting confirmed a concentration-dependent inhibition of HCMV core NEC formation (Figure 5C).

**Figure 6:**
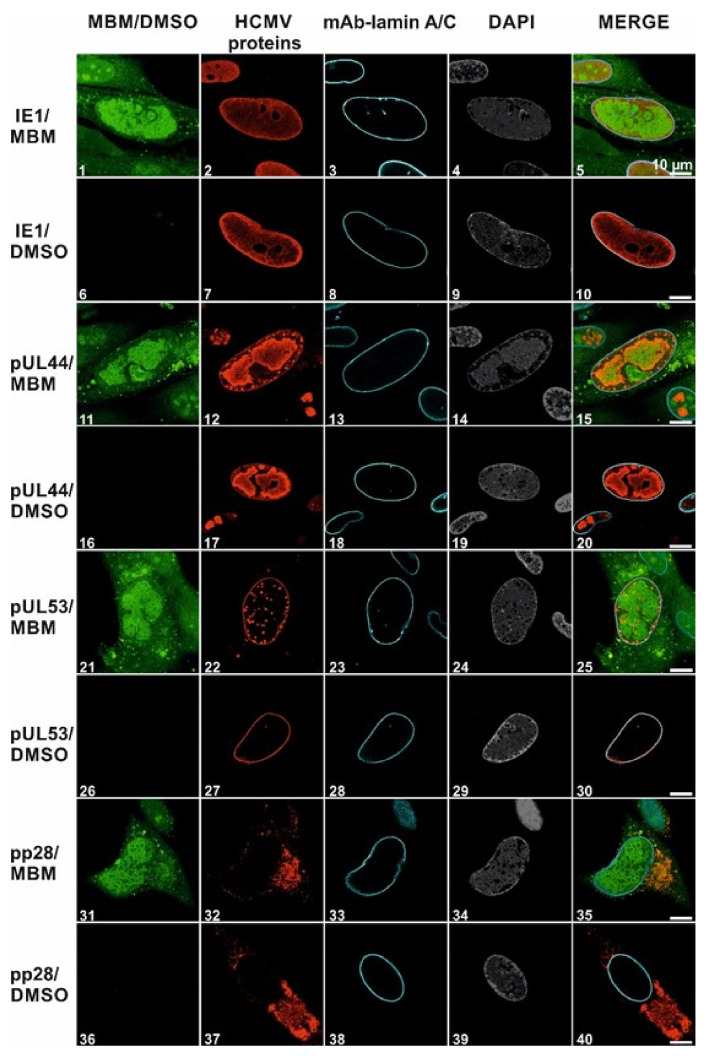
Merbromin does not interfere with the localization of viral proteins other than the core NEC complex in HCMV-infected HFFs: confocal imaging (indirect immunofluorescence staining) of the intracellular localization of representative HCMV proteins (immediate early, IE1; early, pUL44 and pUL53; late, pp28) and cellular lamin A/C in the presence of merbromin (5 µM). Counterstainings of the autofluorescent merbromin and the nuclei (DAPI), as well as a merge of all signals (right column) are shown. Scale bars indicate 10 µm. See Section 2.6 for experimental detail.

Finally, the target selectivity of merbromin for the core NEC, which had been shown in vitro by the lack of inhibitory activity on an unrelated protein–protein interaction (HIV-1 gp120–mAb 447 52D, Figure 3D), was also addressed at the level of infected cells. This was achieved by examining the impact of merbromin on the localization of viral proteins other than pUL53, i.e., immediate early protein (IE1, Figure 6, panels 1–10), early protein (pUL44, Figure 6, panels 11–20), and late protein (pp28, Figure 6, panels 31–40), respectively. pUL53 was included as a positive control (Figure 6, panels 21–30). Interestingly, the localization of none of the other proteins was affected by merbromin at 5 µM, indicating that the antiviral effect of the compound shown in Figure 4 is brought about by an inhibitory effect on the intranuclear formation of the HCMV-specific core NEC, and based on the ability of merbromin to interfere with the pUL50-pUL53 interaction. The target selectivity of merbromin will be further addressed in ongoing and future studies, by investigating its effect on other herpesviruses, as well as related viruses such as adenoviruses.

In a related approach, the antiviral activity of merbromin was investigated by testing its effect on the formation of cytoplasmic viral assembly complexes (cVACs), which correlates with the efficiency of viral nuclear egress. Three different human cell types, i.e., MRC-5, ARPE-19, and HFF, were infected with three different strains of HCMV, i.e., TB40 UL32-GFP, TB40E, and AD169 (Table 1) [11]. In this assay, merbromin was found to dose-dependently reduce the cVAC count for all three viral strains, further substantiating the inhibitory effect of merbromin on HCMV nuclear egress.

## 4. Conclusions 

Screening of the Prestwick Chemical Library^®^ of approved drug compounds yielded small molecule inhibitors of HCMV core NEC formation. These compounds, i.e., verteporfin and merbromin, were found to inhibit the HCMV pUL50-pUL53 interaction at nanomolar concentrations; however, verteporfin was not further analyzed due to its cytotoxicity. The inhibitory effect of merbromin on the pUL50-pUL53 interaction was shown to be both compound- and target-specific. Although merbromin is a potentially toxic organomercuric drug that is no longer in clinical use, its lack of cytotoxicity at the concentrations required for effective core NEC inhibition enabled its evaluation in the context of HCMV infection. The demonstrated ability of merbromin to inhibit HCMV infection of cells, as well as to disrupt HCMV NEC nuclear rim formation, is the first indication of the feasibility to inhibit the pUL50-pUL53 interaction by a small molecule, which is apparently able to be passively taken up by cells, as well as to penetrate the nuclear membrane. Thus, while not being an ideal drug candidate by itself, merbromin may serve as a blueprint for small molecules with high HCMV core NEC inhibitory potential as candidates for novel anti-herpesviral drugs. Ongoing studies are aimed at elucidating the molecular mechanism of inhibition of the pUL50-pUL53 interaction by merbromin, as well as the selectivity of this effect with respect to other herpesviruses and additional viruses. 

## Figures and Tables

**Table 1:** Effect of merbromin on the formation of viral cytoplasmic assembly complexes (cVACs) ^a^.

Conditions of HCMV Infection ^b^		TB40 UL32-GFP on MRC-5	TB40E on ARPE-19	AD169 on HFF
DMSO		88 ± 0.17%	94.3 ± 2.73%	89 ± 1.39%
merbromin	1.25 µM	61 ± 1.24%	79.4 ± 1.29%	71 ± 4.28%
	2.5 µM	51 ± 1.84%	49.5 ± 0.87%	48 ± 0.05%
	5 µM	35 ± 1.28%	27.4 ± 2.38%	32 ± 0.41%
	10 µM	26 ± 0.51%	26.8 ± 0.97%	23 ± 1.01%

^a^ HCMV infection was performed with cells cultivated in 6-well plates on coverslips at a low multiplicity (MOI 0.1) for 5 days. Cells were fixed and used for indirect immunofluorescence staining with an antibody against viral pp150 (pUL32) or for direct microscopic counting of the autofluorescent fusion protein expressed by a recombinant virus (TB40 UL32-GFP). Merbromin was used at the indicated concentration range (1.25–10 µM; DMSO as solvent control), and the formation of pp150-positive cVACs was quantified through visual microscopic counting. All counts were performed in duplicate, and mean values ± standard error are given. ^b^ Three different human cell types and three different viral strains were used as follows: MRC-5, human lung fibroblasts reaching senescence after 45–60 passages; ARPE-19, human retinal epithelial cells; HFF, primary human foreskin fibroblasts; HCMV, recombinant TB40 UL32-GFP fibroblast-adapted; TB40E, epithelial cell-adapted strain; AD169, fibroblast-adapted laboratory reference strain. For primary data, see [11].

## Data Availability

Not applicable.
